# A Numerical Analysis Model for Interpretation of Flow Cytometric Studies of *Ex Vivo* Phagocytosis

**DOI:** 10.1371/journal.pone.0026657

**Published:** 2011-11-04

**Authors:** Ted S. Strom, Praveen Anur, Amanda Prislovsky

**Affiliations:** 1 Department of Pathology and Laboratory Medicine, University of Tennessee Health Sciences Center, Memphis, Tennessee, United States of America; 2 Department of Pathology and Laboratory Medicine, Memphis Veterans Administration Medical Center, Memphis, Tennessee, United States of America; 3 Oregon Health Sciences University, Portland, Oregon, United States of America; Heart Center Munich, Germany

## Abstract

The study of *ex vivo* phagocytosis via flow cytometry requires that one distinguish experimentally between uptake and adsorption of fluorescently labeled targets by phagocytes. Removal of the latter quantity from the analysis is the most common means of analyzing such data. Because the probability of phagocytosis is a function of the probability of adsorption, and because partially quenched fluorescence after uptake often overlaps with that of negative controls, this approach is suboptimal at best. Here, we describe a numerical analysis model which overcomes these limitations. We posit that the random adsorption of targets to macrophages, and subsequent phagocytosis, is a function of three parameters: the ratio of targets to macrophages (m), the mean fluorescence intensity imparted to the phagocyte by the internalized target (alpha), and the probability of phagocytosis per adsorbed target (p). The potential values of these parameters define a parameter space and their values at any point in parameter space can be used to predict the fraction of adsorption(+) and [adsorption(−), phagocytosis(+)] cells that might be observed experimentally. By systematically evaluating the points in parameter space for the latter two values and comparing them to experimental data, the model arrives at sets of parameter values that optimally predict such data. Using activated THP-1 cells as macrophages and platelets as targets, we validate the model by demonstrating that it can distinguish between the effects of experimental changes in m, alpha, and p. Finally, we use the model to demonstrate that platelets from a congenitally thrombocytopenic WAS patient show an increased probability of *ex vivo* phagocytosis. This finding correlates with other evidence that rapid in vivo platelet consumption contributes significantly to the thrombocytopenia of WAS. Our numerical analysis method represents a useful and innovative approach to multivariate analysis.

## Introduction

Ex vivo studies of the phagocytosis of platelets, red cells, and microorganisms are useful for the study of disease states such as autoimmune thrombocytopenia, hemolytic anemia, immunodeficiency, and a number of infectious diseases. While phagocytosis can be reliably distinguished from adsorption by confocal microscopy, that method is not well suited to the analysis of large numbers of events. In flow cytometric studies of phagocytosis of fluorescent targets, “quenching” of adsorbed fluorescent markers with agents like ammonium acetate [1], Trypan blue [2], [3], or proprietary kit reagents [4], has been used to distinguish between uptake and adsorption. However, studies which use these methods rarely show control data demonstrating the effectiveness of quenching. Alternatively, a second fluorescent marker able to quantify cells showing adsorption of the targets is sometimes used to make this distinction [5], [6], [7], [8]. This method in fact distinguishes (1) cells showing adsorption OR (adsorption+phagocytosis) from (2) cells showing phagocytosis only. Because the relative proportions of these two groups will be affected both by the ratio of targets to macrophages and the probability of phagocytosis per adsorbed target, simply ignoring the first group excludes relevant data from the analysis of such experiments. Also, quenching of the fluorescence of internalized targets is often accelerated in the low-pH, protease-rich environment they encounter after phagocytosis. This can result in a phagocytosis(+) population evident only as a ‘bulge’ on the negative population [7], making its quantification problematic. In that context, the distinction between an experimental effect on phagocytosis and an effect on quenching efficiency is not immediately evident. The issue is made more difficult to address by the frequent omission of raw data in published studies utilizing this method.

Here we describe a numerical analysis model which resolves these issues. The model evaluates the concurrent contributions of variation in the target to macrophage ratio, the probability of phagocytosis per target, and the fluorescence intensity imparted to the macrophage per internalized target. By experimentally manipulating these three variables, we demonstrate that the model correctly attributes changes in the resultant data to changes in those variables. We then go on to use the model to assess the probability of phagocytosis of platelets from patients with the Wiskott-Aldrich Syndrome (WAS), an X-linked recessive condition characterized by a severe thrombocytopenia.

## Results

**Figure 1:**
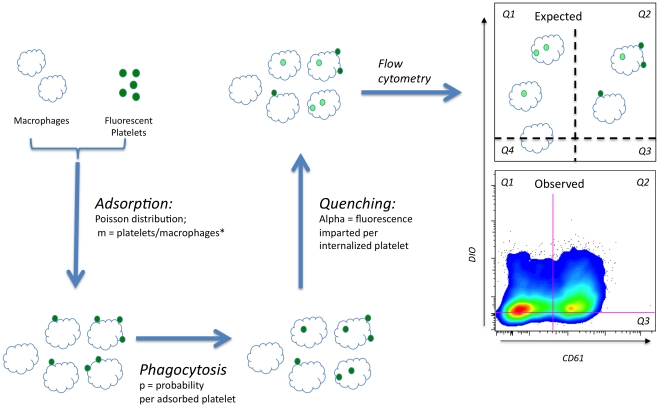
Experimental design and modeling. Platelets are labeled with DIO and exposed to macrophages (THP-1 cells) via a low speed centrifugation step, then incubated at 37 degrees. The processes of adsorption, phagocytosis, and quenching of the fluorescence of internalized platelets are shown schematically, with reference to the parameters used in numerical modeling. The asterisk denotes that “m” is the *functional* ratio of platelets to macrophages (see text). Cells are then exposed to PE-anti-CD61, and analyzed by flow cytometry. The expected results (top right) include cells showing variable numbers of internalized platelets, adsorbed platelets, and mixtures of the two. Typical observed results for normal control platelets are also shown (bottom right).

The experimental design we used to assess the ex vivo uptake by macrophages of platelets from WAS patients and normal controls is shown schematically in **figure 1**, as are results obtained from a representative control experiment. We chose to label platelets with the lipophilic marker DIO (rather than the more commonly used amine-reactive marker CMFDA) because this increases the sensitivity of the assay markedly, allowing useful results to be obtained with as few as 3 million platelets per well (vs. 30 million CMFDA labeled platelets [5]). This improvement was needed to allow study of platelets from thrombocytopenic patients, and presumably results from reduced quenching of the fluorescence of internalized platelets. DIO has been used by others for in vivo red cell turnover studies [9].

The gates in figure 1 are arbitrarily drawn to place 50% of the negative control population in quadrant 1. The purpose of this gating scheme is discussed below. In a typical experiment, quadrant 1 is assumed to contain both (adsorption(−), phagocytosis(−)) and (adsorption(−)phagocytosis(+) cells. All of the adsorption(+) cells are contained in quadrants 2 and 3. The data suggests that both adsorption and phagocytosis occur, but simple quantification of the data in each quadrant (or any similarly drawn quadrants) does not directly address the question of how much of each is taking places.

### Numerical analysis model

Our hypothesis is that optimal values of three parameters can be found which predict, via the numerical modeling described below, the observed results. The parameters are m (the functional ratio of platelets to macrophages); p (the probability of phagocytosis of an adsorbed platelet); and alpha (the fluorescence intensity imparted to the macrophage by phagocytosis of one platelet). M is not equivalent to the input ratio of platelets to macrophages in each study, since not all of the platelets used in each assay are expected to come into contact with macrophages. It is instead assumed to be proportional to that ratio.

### Assumption I

We assume that our experimental method initially generates a set of macrophage populations showing *n* adsorbed platelets (n = 0 to n = j) for a given value of m. It follows that *m* determines the probability of generation of a set of macrophages demonstrating *n* adsorbed platelets (P(n)) via a random (poisson) distribution:
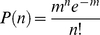
(1)It follows that the probability of a macrophage receiving one or more adsorption “hits” (P(n>1) is:
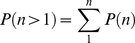
(2)Within reasonable limits of m (<6.5), this can be evaluated numerically over a range of n values from 1 to 12 (yielding a net cumulative probability for 12 or less occurrences of over 0.98).

### Assumption II

P is assumed to be independent of the number of adsorbed platelets per macrophage. There are to our knowledge no known pro- or anti-phagocytic signaling pathways which dominate the outcome of platelet-macrophage interactions in such a way as to undermine this assumption.

It follows from *assumption II* that random phagocytosis occurs after adsorption within each such population at a probability per adsorbed platelet of P. For each population defined by n hits, the probability that a given cell will demonstrate only phagocytosis (and no adsorbed platelets) at the end of the study is therefore:
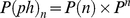
(3)


### Assumption III

Alpha (normalized to the standard deviation of the negative control population) is assumed to be the geometric mean of a lognormal distribution of fluorescence intensities with the same standard deviation (SD) as the negative control population. This is based on the relatively weak fluorescence intensity imparted by phagocytosis in our system (the positive population is not distinct from the negative one – **figure 1**). Platelet labeling with DIO, like that imparted by fluorescent antibodies, is lognormal (data not shown).

It follows from these assumptions that the observed fluorescence intensity histograms for adsorption(−) cells can be modeled as the sum of multiple histograms defined by a range of values of n, multiples of alpha, and common values of SD. This is expected to yield the lognormal distribution of fluorescence intensities we see in a typical experiment.

**Figure 2:**
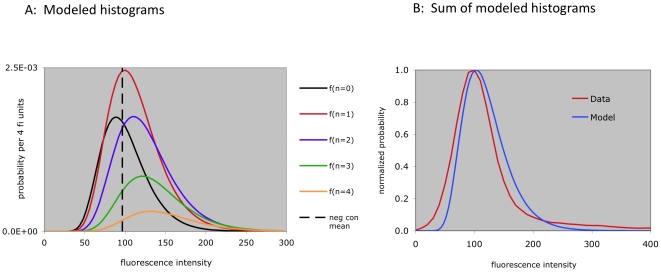
Observed and modeled fluorescence intensity histograms. A) Modeled histograms for n = 0 to n = 4. Empirically chosen parameter values (see text) are p = 0.739, alpha = 2.53, m = 2.17. Mean and SD values of the log transformed negative control data are 4.57 and 0.29. B) The sum of the predicted fluorescence intensities for n = 0 to n = 12 is shown in blue. Data for comparison (CD61 negative cells, figure 1) is shown in red.

For a given set of parameter values, a (measured) negative control population fluorescence intensity geometric mean, and a (measured) SD value, we can therefore generate (in a spreadsheet) a predicted distribution of fluorescent, adsorption(−) populations (one for each value of n). An example is shown in **figure 2**. These populations can in turn be summed over the range n = 1 to n = 12, to generate a predicted histogram of fluorescence intensities. Comparison of one such histogram (generated from empirically selected parameter values) to observed data shows that it is possible to use this model to generate predicted results which approximate observed flow cytometric data (**figure 2B**). We note, however, that the predicted findings do not match perfectly with the high fluorescence intensities seen in a small fraction of the cells in this case (see discussion).

It follows that the predicted fraction of adsorption(+) cells is given by:
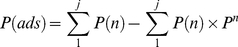
(4)Equation (4) predicts, for a given set of parameters m, p, and alpha, the type of results we experimentally quantify in quadrants 2 and 3 (**figure 1**). To predict the fraction of cells falling in quadrant 1, we use the (measured) mean fluorescence intensity and standard deviation of the negative control population (FI(neg)). It follows from *assumption III* that for each phagocytosis(+), adsorption(−) population originally receiving n adsorption ‘hits’, the fraction of cells which show a fluorescence intensity above the mean of the negative control is determined by a lognormal distribution of FI with a mean of FI(neg)+n*alpha. Graphically, these are the values to the right of the dashed vertical line in **figure 2A**. The sum of these values over the range (n = 0 to n = 12) predicts the fraction of cells in quadrant 1 (Q1, **figure 1**). (Use of the mean of the negative control cells as a reference point is the reason the horizontal line of demarcation in **figure 1** was placed at that level).

### Optimal parameter search method

To find values of m, p, and alpha which best fit the observed data for any given experiment, we define the following terms:

**PS** (parameter space): the set of points defined by p(i = 0 … n), alpha(j = 0 … n), m(k = 0 … n).
**Q1** = the fraction of observed cells in quadrant 1 (**figure 1**)(adsorption(−), phagocytosis(+/−))
**Q(2+3)** = the fraction of observed cells in quadrants (2+3)(**figure 1**) (adsorption(+))
**Q1(p)** = the predicted fraction of cells in quadrant 1
**Q(2+3)(p)** = the predicted fraction of cells in quadrants (2+3)
**R1** = |Q1−Q1(p)|/Q1, the (residual) difference between observed and predicted values of Q1 for point p(i), alpha(j), m(k).
**R2** = |Q(2+3)−Q(2+3)(p)|/Q(2+3), the (residual) difference between observed and predicted values of Q(2+3) for point p(i), alpha(j), m(k).
**Rt** = R1+R2

R1, R2, and Rt can be evaluated for any set of parameter values m, p, and alpha in comparison to any set of data. Our hypothesis is that numerical evaluation of a range of possible parameter value sets in parameter space will allow us to identify one or more minimal local values of Rt (Rt(local)). These in turn will correspond to optimal values of m, p, and alpha for the data set evaluated.

To achieve this, we designed a simple excel spreadsheet consisting of 400 repeated “blocks”. Each block performs the above calculations for a given set of parameter values. Twenty blocks evaluate a user-defined range of P values (a line in parameter space, or a “set”). Twenty sets evaluate that range of P values over a user defined set of alpha values (a plane in parameter space). The spreadsheet is then instructed to repeat the entire process for 20 user-defined values of m (20 planes), evaluating a total of 8000 points in parameter space. The process can be directed to evaluate multiple adjacent volumes of parameter space as needed.

To evaluate the results obtained from such a set of calculations, we define the following terms:

R1min(j,k) = the minimum value of R1 for PS points (i = 0 …1, j, k)
R2min(j,k) = the minimum value of R2 for PS points (i = 0 …1, j, k)
Rtmin(j,k) = the minimum value of (R1min+R2min) for PS points (i = 0 …1, j, k)
P1min(j,k) = the P value (i) corresponding to R1min
P2min(j,k) = the P value (i) corresponding to R2min
**Ptmin(j,k) = the P value (i) corresponding to Rtmin**

Similarly, Rtmin for a plane defined by m = k, and a set of values of p and alpha is termed Rtmin(k).

It is then necessary to define reasonable boundaries for parameter space. P values necessarily lie between 0 and 1. M values are limited to m<6.5 due to the limited range of n values evaluated in each block (as noted above). We found that alpha values above 4.5 tend to generate, for values of p and m in the above ranges, two or more populations in quadrant 1. As our studies in most cases do not generate such a distinct population (see, for example, **figure 1**), we did not evaluate alpha values above 4.5.

### Utility of the model

**Figure 3:**
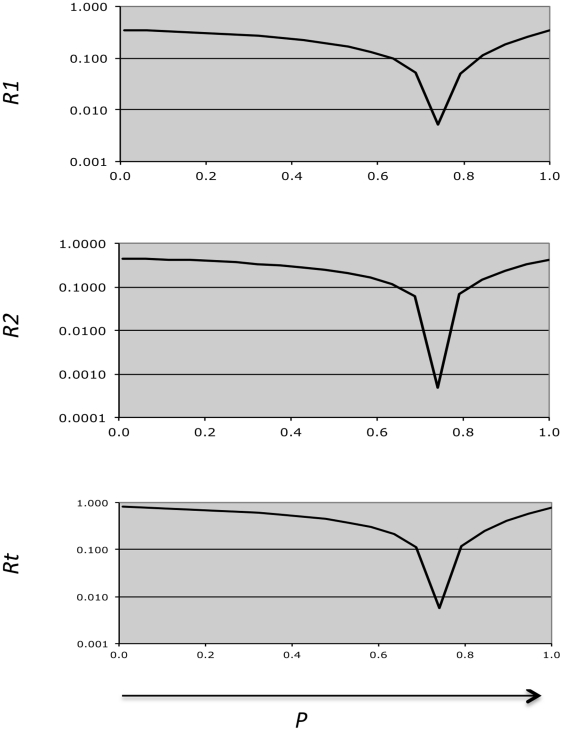
Assessment of a series Rt values (p-axis). Using empirically chosen m and alpha values, and a series of p values, predicted Q1 and Q(2+3) values were compared to observed values for the data shown in figure 1. P value resolution is 0.04 (20 evaluated points). A single Rtmin(j,k) value is identified.

The identification of optimal parameter values using this model begins with identification of Rtmin(j,k). An example is shown in **figure 3**, for data obtained with normal control platelets and values of M and alpha which were chosen empirically. Residuals (R1, R2, and Rt) are in this case calculated for a series of P values, and an optimal P value is evident at the minimum Rt value shown in the figure. The sum of the predicted Q1 and Q(2+3) values (**table 1**) is in this case within 0.8% of the (summed) experimental data.

**Table 1:** Data and parameter values for figure 3.

	observed (mean)	predicted at Rtmin(j,k)
Q1	0.408	0.405
Q(2+3)	0.427	0.432
m		2.18
alpha		2.53
Ptmin(j,k)		0.739

The m and alpha values used, and the p value corresponding to the minimum Rt value (Ptmin(j,k)), are shown.

**Figure 4 pone-0026657-g004:**
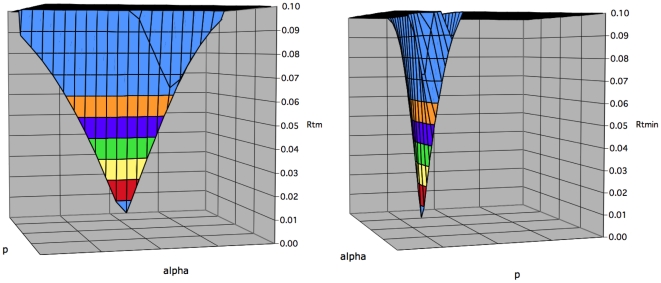
Assessment of a series of Rtmin(j,k) values (alpha axis). Rt was evaluated for the data shown in figure 1 in an empirically chosen plane defined by M = 2.18. Alpha values assessed ranged from 1 to 4 (resolution 0.15). P values were assessed (for each alpha value) as in figure 3. Left: A single local Rtmin value (Rtmin(k)) is identified in this plane. Right: The graph is rotated 90 degrees on the z axis.

Evaluation of Rtmin(k) is then undertaken by repeating this process for a range of alpha values, generating (for an empirically chosen value of m) the Rtmin(j,k) values shown in **figure 4**. Here again, a single Rtmin(k) value is evident at this value of m.

**Figure 5 pone-0026657-g005:**
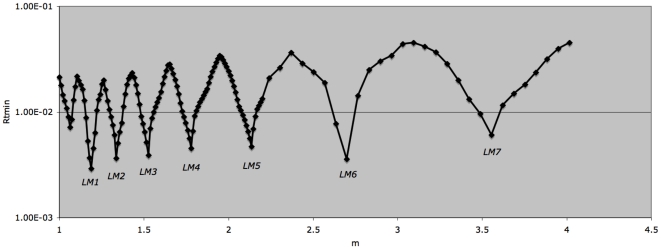
Assessment of a series of Rtmin(k) values (m axis). A series of planes (defined by m values) was evaluated as shown in figure 4. P and alpha resolutions were as described in figures 3 and 4. The alpha range was manually adjusted to follow the local minima (LM) evident at lower resolution scans; alpha values for the local minima shown ranged from 4.05 (LM1) to 1.06 (LM7). M resolutions were 0.01 (range m = 1 to m = 2.25) and 0.0625 (m = 2.25 to m = 4).

Finally, a range of m values is evaluated. The Rtmin(k) values from each (for the data in **figure 4**) are shown in **figure 5**. Multiple local minima (LM) are evident, each diverging from the data by less than 1%. The predicted fluorescence histogram for LM5 is shown in figure 2. Predicted histograms for LM1, 3, and 7 are shown in figure S1. Parameter values for the local minima are shown in table S1. Although a single, ‘global’ Rtmin value was sought, the multiple local minima found remain useful (see below).

The resolving power of the model was evaluated over multiple ranges of m, p, and alpha values. Although m could be usefully parsed into values as fine as 0.01 (**figure 5**), we found empirically that “high resolution” studies at p value resolutions below 0.04 and alpha value resolutions below 0.1 generated multiple, narrowly spaced local minima analogous to ripples on larger wave patterns (data not shown). Such studies were not pursued further.

We next systematically altered experimental conditions in a manner expected to affect, separately, each of the three parameter values, and asked whether the model interpreted the resultant data in the expected way. Specifically, we altered the fluorescence intensity of the platelets (A) and the ratio of platelets to macrophages (B), changes expected to alter the predicted optimal values of alpha and m, respectively. To increase the probability of phagocytosis, we opsonized the platelets with anti-CD61 antibody (C).

**Figure 6:**
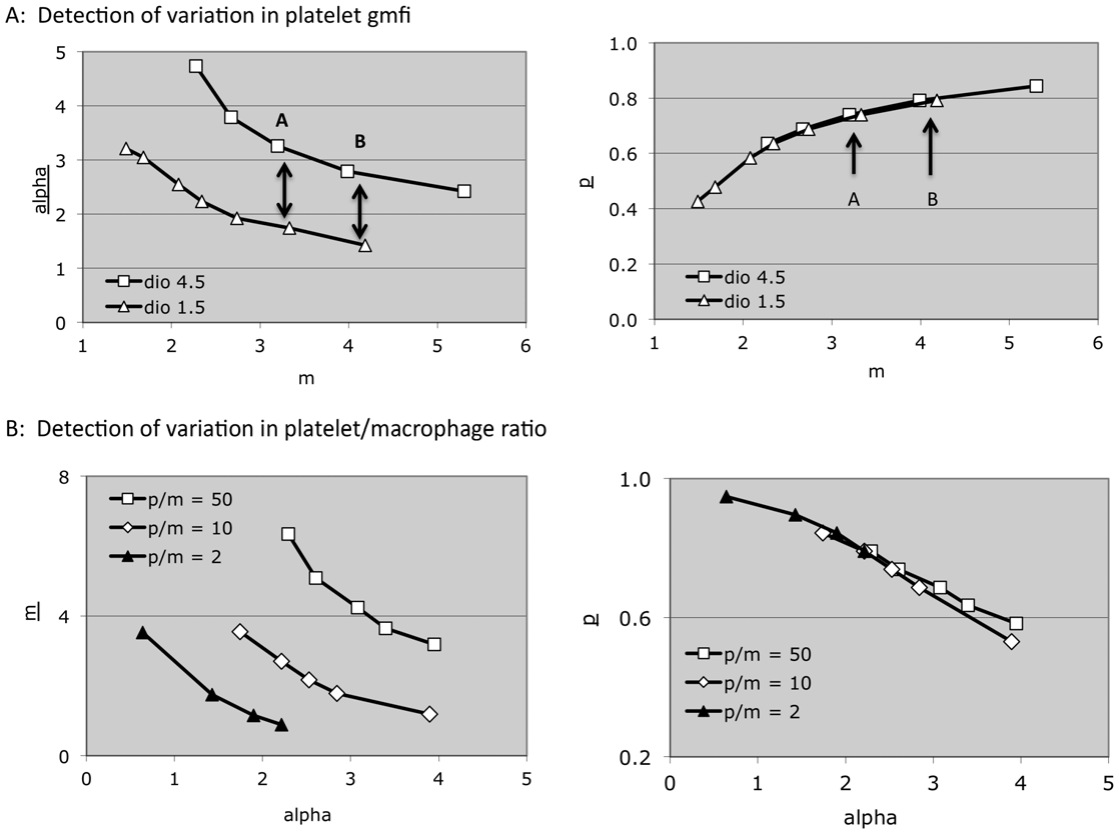
Effects of varying key input values on local Rt minima. A) Normal control platelets were labeled with DIO at the uM concentrations shown, resulting in geometric mean fluorescence intensities of 2796 and 1473, respectively (arbitrary units). Evaluation of parameter space was performed as in figures 3 to [Fig pone-0026657-g004] 5, at resolutions p = 0.04, alpha = 0.15, and m = 0.0625. Local Rtmin(k) points in parameter space were identified as in figure 5. B) Normal control platelets were labeled at 1.5 uM DIO, and exposed to activated THP-1 cells at the platelet/macrophage ratios shown. Parameter space evaluation was performed as in (A).

For case (A), our prediction is that the model would interpret the resulting data as showing an increase in alpha and not an increase in p, The resulting local Rt minima (identified as in **figure 5**) verify these predictions. Specifically, while the model does not tell us which m value is optimal for either data set, it does allow us to compare results obtained at a constant platelet-to-macrophage ratio (hence, a constant m value). We do this by comparing those minima with the most similar m values, such as the pairs designated A and B in **figure 6A**. The figure shows that alpha values for these pairs are substantially increased when the platelets used in the assay were labeled at a higher fluorescence intensity, while p values are unaffected.

**Figure 7:**
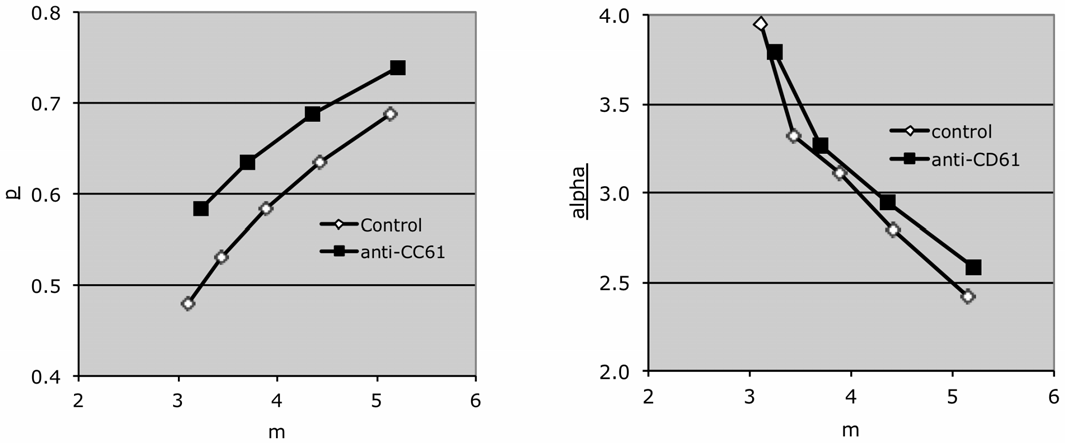
Effect of antibody opsonization on local Rt minima. Control platelets were exposed to anti-CD61 antibody (50 ng per million platelets) or to an isotype control antibody, then exposed to activated THP-1 cells. Evaluation of parameter space was performed as in figures 3–6.

Similarly, in case (B), while the model does not tell us which alpha value is optimal, it does allow us to compare results obtained at a constant platelet fluorescence intensity (hence, a constant alpha value), using the same nearest neighbor method just described. The minima used for nearest neighbor comparisons (**figure 6b**) show clearly that data obtained by changing the platelet to macrophage ratio is interpreted by the model as an increase in m and not in p. For case (C), data obtained with platelets opsonized with anti-CD61 was interpreted by the model as demonstrating an increased p value and not an increase in alpha (**figure 7**). Fc-receptor mediated uptake of opsonized platelets by THP-1 cells has been previously described [3].

**Figure 8:**
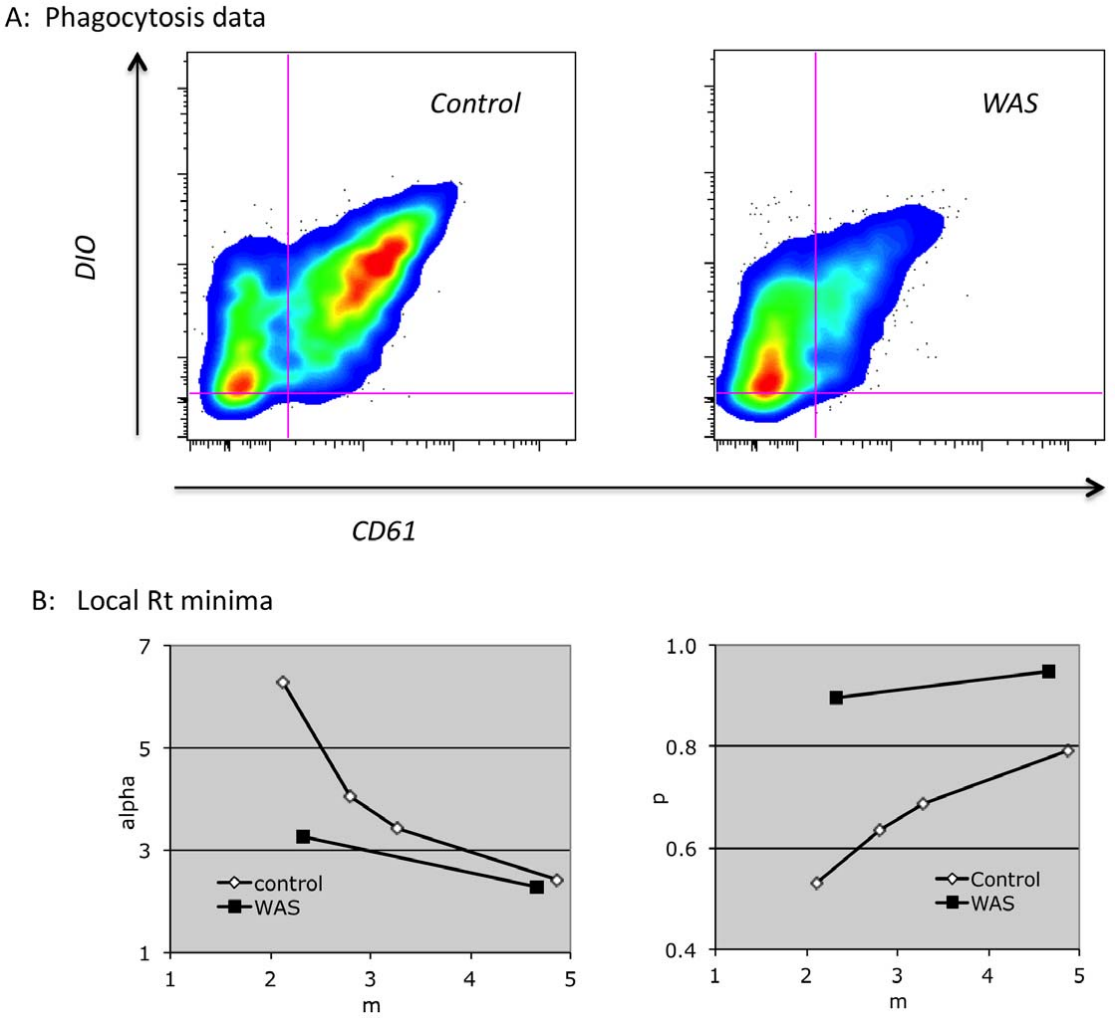
Effect of platelet WASP deficiency on phagocytosis. A) DIO-labeled platelets from an adult control, or from a WAS patient, were exposed to THP-1 cells and analyzed as described in figure 1. B) Parameter space was evaluated as in figures 3 to [Fig pone-0026657-g004] 5, in comparison to mean values of duplicates of the phagocytosis assays shown in (A). Local Rt minima are shown.

Application of the model to data obtained from a WAS patient is shown in **figure 8**. Qualitatively, the data suggests a significant increase in the probability of phagocytosis of patient vs. control platelets, as the fraction of cells in quadrant 1 vs. 2 is markedly increased. One could conceivably simply ignore the data in quadrants 2 and 3, and argue that comparison of the quadrant 1 values implies an increased rate of phagocytosis of WAS platelets. Aside from the problem of excluding half of the data, this interpretation is limited by the higher fluorescence intensity demonstrated by the WAS platelets in this study (1531, vs. 604 for the control platelets (arbitrary units)), which could have contributed to the large number of cells in quadrant 1 (WAS). Modeling results (**figure 8B** and **table 2**), however, allow comparison of the WAS local Rt minima to the two control minima demonstrating similar m values (the first and fourth control minima). Both comparisons show that the probability of phagocytosis is indeed increased for the WAS platelets. Concerning alpha, comparison of the local minima at low m values suggests that it is reduced, while comparison of the minima at high m values suggests it is comparable to that of the control platelets. Given the higher fluorescence intensity of the WAS platelets used in the study, a negative effect of platelet WASP deficiency on the quenching of internalized platelets is likely.

**Table 2:** Predicted and observed results for control vs. WAS platelet phagocytosis.

		observed	predicted (1)	predicted (2)
control	Q1	0.327	0.310	0.325
control	Q(2+3)	0.637	0.630	0.638
control	Rtmin		0.024	0.002
WAS	Q1	0.698	0.695	0.704
WAS	Q(2+3)	0.218	0.215	0.217
WAS	Rtmin		0.005	0.007

Q1 and Q(2+3) values for the WAS local Rt minima shown in figure 8 are compared to the control minima with the nearest m values (the first and fourth control minima in figure 8B).

## Discussion

While reduction of experimental conditions to the study of individual variables is a universal objective in the biological sciences, its infeasibility in some types of experiment does not necessarily prevent interpretation. The dysregulation of phagocytosis plays a key role in a number of disease processes, but tools for its study *ex vivo* often do not allow all relevant variables to be precisely controlled. In the case of WAS, for example, platelets are difficult to obtain because the condition is rare and the patients are severely thrombocytopenic. Low platelet yields in turn make control of variables like their fluorescent labeling intensity difficult. Direct assessment of differences in the quenching of that fluorescence after phagocytosis is still less tractable, and has not even been attempted in other published phagocytosis studies.

Here we have used numerical analysis of multiple variables to leapfrog these experimental difficulties. After exposure of macrophages to fluorescent target platelets, we divided the resultant fluorescent macrophage populations (**figure 1**) into those showing adsorbed platelets (Q2+3) and those adsorption(−) cells showing a mixture of phagocytosis(+) and phagocytosis(−) cells (Q1). Using a simple excel spreadsheet, we predicted the fraction of cells expected to be distributed in (Q1) and (Q2+3), based on three parameter values (m, p, and alpha). By scanning all available combinations of the latter values in a parameter-defined volume (parameter space), and evaluating the difference between the resultant predictions and actual data, we were able to arrive at optimal parameter set values for that data. Two or more such optima (local residual minima) were found for each data set, and our analysis did not allow us to determine a “global” minimum for any data set. However, control of appropriate experimental variables allowed us to match local minima for control and test data, and to distinguish between the effects of altered platelet/macrophage ratios, of altered platelet fluorescence, and of an altered probability of phagocytosis.

The ability of the model to distinguish between a series of controlled experimental effects on individual parameter values indicates that the assumptions on which the model is based are functional. The model succeeds in predicting both the fraction of adsorbed platelets and the fraction of platelets taken up by macrophages, to within 1% of the observed values in most cases. However, its predicted fluorescence histograms typically under-estimate the fraction of cells showing high fluorescence intensities (**figure 2**). Among the factors which could contribute to this are platelet dimerization or multimerization; an effect of n (the number of adsorbed platelets per macrophage) on p; a distributed rather than constant capacity for phagocytosis in the macrophage population; and an effect of n on the standard deviation of alpha.

Finally, preliminary results from a patient with a congenital thrombocytopenia (the Wiskott-Aldrich Syndrome, or WAS) indicate an increased susceptibility of these platelets to ex vivo phagocytosis. The model allowed us to distinguish in this case between the effects of variation in the fluorescence intensity imparted by phagocytosis (alpha) and variation in the probability of phagocytosis per macrophage-adsorbed platelet. The apparent reduction in the quenching of the fluorescence of internalized WAS platelets might be expected due to the smaller volume of these platelets.

We cannot rule out the possibility that adsorption of platelets to macrophages is impaired by platelet WASP deficiency. This might result in a lower m value for WAS vs control platelets, despite identical input ratios of platelets to macrophages. This could not explain our findings, however, because both of the WASP(−) minima seen in figure 8B demonstrate a higher p value than any of the control minima - a finding we have replicated with specimens from other WAS patients (Prislovsky et al., manuscript in preparation).

Our results suggest that accelerated in vivo platelet phagocytosis could contribute significantly, via increased platelet turnover, to the thrombocytopenia of WAS. We have previously demonstrated rapid in vivo turnover of WASP(−) platelets in a murine model of WAS [5], rapid ex vivo phagocytosis of antibody opsonized murine WASP(−) platelets, and a greater effect of opsonization on the in vivo clearance of WASP(−) vs. WT platelets [10]. Our studies of the effects of opsonization on human WASP(−) platelets will be reported separately (Prislovsky et al., manuscript in preparation).

The advantage of this model over other methods of interpreting this type of data lies in its incorporation of all the data (including quantification of cells showing adsorption), and its ability to distinguish between experimental effects on target quenching and phagocytosis rate. As such it adds significantly to our ability to study phagocytosis ex vivo. In broader terms, by taking into account data which might otherwise be considered uninterpretable due to the simultaneous effects of more than one variable, the model demonstrates a practical and unique numerical approach to multivariate analysis.

## Materials and Methods

### Reagents

Mouse anti-Human CD61 (clone VP-PL2), Mouse anti-Human CD41 (clone HIP8), PE labeled mouse anti-human CD61, and PE labeled Mouse anti-human CD41 were obtained from BD Biosciences. Dimethyl sulfoxide (anhydrous), Phorbol 12-myristate 13 acetate (PMA), Prostaglandin E1 (PGE1), L-glutamine, and Hank's Balanced Salt solution were purchased from Sigma (St. Louis, MO). RPMI media, trypsin (catalog number 25300-054), beta mercaptoethanol, 3,3′-dioctadecyloxacarbocyanine perchlorate (DIO, catalog number D275), and penicillin/streptomycin were purchased from Invitrogen/Life Technologies. Fico/lite for platelets was from Atlanta Biologicals. THP-1 (TIB-202) cells were purchased from ATCC.

### Human platelet preparation

All studies were approved by the Institutional Review Boards of the Memphis VA Medical Center and the University of Tennessee Health Science Center. Informed consent of the participants or (for minors) their parent/guardian was obtained in all cases. All clinical investigation was conducted according to the principles expressed in the Declaration of Helsinki. Citrate-anticoagulated blood was obtained from healthy volunteer adults. For studies comparing controls to WAS patients, both specimens were placed on a rotating platform immediately after blood was obtained. WAS patient specimens were shipped overnight on such a platform, while control specimens were stored at room temperature on such a platform. Specimens were layered over a ficoll cushion (Fico/lyte) and centrifuged at 350 g for 15 minutes at room temperature. The platelet layer was diluted in modified tyrode's buffer (20 mM Hepes, 137 mM NaCl, 13.8 mM NaHCO_3_, 2.5 mM KCl, 0.36 mM NaH_2_PO_4_-H_2_O, 5.5 mM glucose, 0.25% bovine serum albumin, 1 mM MgCl_2_) supplemented with 1 ug/ml of PGE1, and centrifuged at 6000 g for 5 minutes. Pellets were resuspended in modified tyrode's buffer supplemented with PGE1, and counted with a Beckman Coulter model Z2 particle analyzer.

### DIO labeling of platelets

Platelet preparations were labeled by mixing them 1∶1 with a solution of pre-warmed DIO (1.0 uM), Tween 20 (0.7 mM) and DMSO (92%). Final platelet concentrations in the reaction ranged from 9×10^4^ to 8×10^5^ per ul. The reaction was allowed to proceed for 30 minutes at 37 degrees in darkness. After addition of 5 volumes of modified tyrode's buffer supplemented with PGE-1, platelets were left on a rotating platform for 10 minutes at room temperature in darkness (to facilitate removal of DMSO), centrifuged at 6000 g for 5 minutes at room temperature, and resuspended in modified tyrode's buffer supplemented with PGE-1.

### Antibody binding

To facilitate comparison to concurrent studies of the effects of antibody opsonization, platelets were in all cases exposed to either an opsonizing antibody or an isotype control antibody as follows. Antibodies were bound (after DIO labeling) for 2 hour at room temperature on a rotating platform protected from light. Five volumes of modified tyrode's buffer supplemented with PGE1 was added, platelets were centrifuged at 6000 g for 5 minutes at room temperature, and platelets were resuspended in 10% IFBS RPMI media.

### THP-1 cells

THP-1 cells were grown in 10% IFBS RPMI supplemented with Penicillin/streptomycin, L-glutamine, 5 um beta mercaptoethanol, and 10% inactivated fetal bovine serum (IFBS). Cells were activated for 3 hours 37 C with 50 ng/ml of PMA at a concentration of 1×10^6^ cells/ml. After activation, cells were centrifuged 450 g for 10 minutes at room temperature, then resuspended in the above medium at 3×10^5^ cells/ml, and distributed in 48 well dishes. The majority of the cells remain non-adherent at this point, with some showing loose adherence to the dish.

### Phagocytosis

Platelets were added to wells containing activated THP-1 cells at 10 platelets/cell, using duplicate or triplicate wells for each experimental condition studied. Cells and platelets were centrifuged for 1 minute at 200 g, room temperature, then placed in a 37 degree, 5% CO2 incubator for 1 hour. The majority of the cells were non-adherent at this point. They were collected by centrifugation of the supernatant at 400 g for 10 minutes at 4°C. Cells remaining on the dish were rinsed 3 times with HBSS, treated with trypsin for 15 min 37 C, centrifuged at 400 g for 10 minutes at 4°C, resuspended in RPMI/10% IFBS, and pooled with the supernatant cells. The cells were centrifuged again as above, and resuspended in PBS containing PE labeled mouse anti-human CD-61. Cells were Incubated for 30 min at 4°C in the dark, then analyzed with a Becton Dickinson LSRII flow cytometer. Quantification of the mean adsorption(+)(Q2+3) and adsorption(−), phagocytosis(+/−)(Q1) populations was performed as described in the text, using Flowjo software (Treestar, Inc).

### Computation

The numeric analysis model was constructed using Microsoft Excel. For comparison to predicted histograms, flow cytometry data was exported from flowjo as scale values. “Negative” fluorescence values, a consequence of automated baseline correction by the flow cytometer, were corrected by addition of a constant equal to the lowest value in an individual file. Calculations were performed on standard iMac desktop computers.

## Supporting Information

Figure S1 **Data and modeled fluoresence histograms from** **figure 5** **.** Histograms were generated as shown in figure 2, using the parameter values in table S1.
(TIFF)

Table S1 **Parameter values and associated values for the data in** **figure 6** **.**
(DOCX)
