# Supplementary figures and images for: A Numerical Analysis Model for Interpretation of Flow Cytometric Studies of Ex Vivo Phagocytosis
Source: PLoS One. 2011 Nov 4;6(11):e26657. doi: 10.1371/journal.pone.0026657 (PMC3208553; doi:10.1371/journal.pone.0026657)

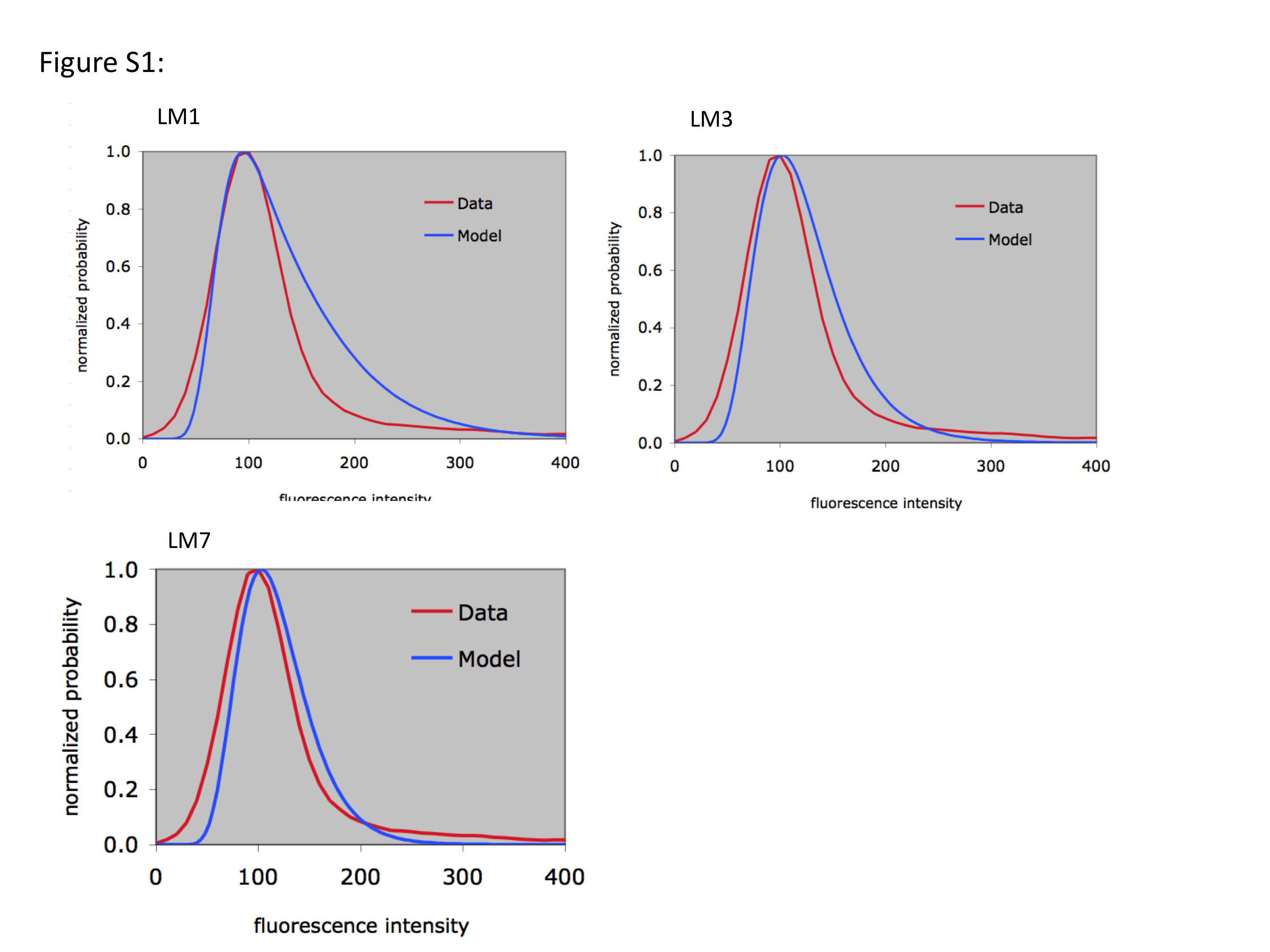

Supplement: Figure S1 — Data and modeled fluoresence histograms from figure 5 . Histograms were generated as shown in figure 2, using the parameter values in table S1. (TIFF) [file pone.0026657.s001.tiff]
