# Supplementary material for: A Numerical Analysis Model for Interpretation of Flow Cytometric Studies of Ex Vivo Phagocytosis
Source: PLoS One. 2011 Nov 4;6(11):e26657. doi: 10.1371/journal.pone.0026657 (PMC3208553; doi:10.1371/journal.pone.0026657)
Supplement: Table S1 — Parameter values and associated values for the data in figure 6 . (DOCX) [file pone.0026657.s002.docx]

Supplemental material:


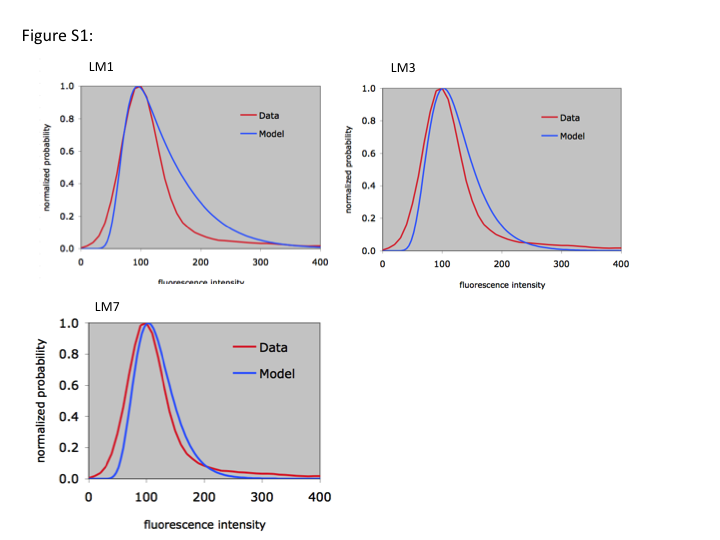


Figure S1: Data and modeled fluoresence histograms from figure 5. Histograms were generated as shown in figure 2, using the parameter values in table S1.

Table S1: Parameter values and associated values for the data in figure 6.
